# Structural Characterization and Immunomodulatory Activity of a Novel Mannoglucogalactan from *Tremella aurantialba*: Implications for Natural Immunotherapy

**DOI:** 10.3390/foods14234126

**Published:** 2025-12-02

**Authors:** Yuemou Zhao, Wenyu Liang, Huaqun Chen, Jinwen Huang, Longyan Zhao, Qingxia Yuan

**Affiliations:** Guangxi Key Laboratory of Marine Drugs, University Engineering Research Center of High-Efficient Utilization of Marine Traditional Chinese Medicine Resources, Guangxi, Institute of Marine Drugs, Guangxi University of Chinese Medicine, Nanning 530200, China; z15132400557@163.com (Y.Z.); 15131121002@163.com (W.L.); 15695648507@163.com (H.C.); huangjinwen1127@163.com (J.H.)

**Keywords:** mushroom, *Tremella aurantialba*, polysaccharide, structural characterization, immunomodulatory activity

## Abstract

Heteropolysaccharides, the principal bioactive constituents of the esteemed medicinal food *Tremella aurantialba*, remain poorly understood in both structure and function. Herein, we describe a novel heteropolysaccharide, designated TAP-2a, isolated from the fruiting bodies of *T. aurantialba* via multi-step column chromatography. With a molecular weight of 16.95 kDa, TAP-2a is dominated by the pyranose forms of ᴅ-galactose (ᴅ-Gal*p*), ᴅ-glucose (ᴅ-Glc*p*) and ᴅ-mannose (ᴅ-Man*p*), accompanied by minor proportions of ᴅ-xylose (ᴅ-Xyl*p*), ʟ-fucose (ʟ-Fuc*p*) and glucuronic acid. Methylation-GC-MS and exhaustive 1D/2D NMR analyses revealed a backbone assembled from →6)-α-Gal*p*-(1→, →6)-β-Glc*p*-(1→, and →3)-α-Man*p*-(1→residues, branched at →2,6)-β-Gal*p*-(1→, →3,6)-α-Gal*p*-(1→, and →2,3)-α-Man*p*-(1→residues, and terminated by β-Glc*p*-(1→, α-Fuc*p*-(1→, and β-Xyl*p*-(1→. This intricate glycosidic architecture generates an exceptionally complex mannoglucogalactan in which a Gal→Man domain is substituted at *O*-3 of Gal by t-β-Glc*p* side chains and at *O*-2 of Man by t-α-Fuc*p* stubs; additionally, a discrete fragment comprising t-β-Glc*p*-(1→3)-β-Glc*p*-(1→ was identified, along with a minor branch in which t-β-Xyl*p* is attached to *O*-2 of a mannose residue. Functionally, TAP-2a proved to be a potent immunomodulator, markedly enhancing the secretion of nitric oxide, interleukin-1β, interleukin-6 and tumour necrosis factor-α while concurrently up-regulating the corresponding mRNA transcripts and augmenting phagocytic capacity. These findings establish the highly elaborate heteropolysaccharides of *T. aurantialba* as powerful immunomodulators that underpin the fungus’s renowned medicinal efficacy.

## 1. Introduction

Polysaccharides are ubiquitous in plants, animals, and microorganisms [1], and have attracted intense interest for their broad therapeutic portfolio—antitumor [2], antidiabetic [3], anticoagulant [4], immunomodulatory [5], anti-inflammatory [6]—combined with low toxicity. Immune homeostasis is the cornerstone of host defense, and numerous polysaccharides act as “immunomodulators” by engaging pattern-recognition receptors to orchestrate cellular and molecular cascades that fine-tune immunity. Edible and medicinal mushrooms are particularly rich in these macromolecules; with mounting pre-clinical evidence and growing public demand for immune enhancement [7,8,9,10], there is an urgent need to translate them into new drugs, dietary supplements, functional foods, and nutraceuticals.

To date, lentinan, *Poria cocos* polysaccharide, *Astragalus* polysaccharide, schizophyllan, and krestin have been approved for clinical use as immunomodulators [11,12]. Beyond these established drugs, a variety of mushroom polysaccharides are also marketed globally as dietary supplements, functional foods, and nutraceuticals [13]. Commercial products are overwhelmingly homoglycans whose simple, repeating structures are readily defined; conversely, potent heteropolysaccharides remain structurally opaque, hampering mechanistic insight and further development. Precise structural elucidation of these complex glycans is therefore a prerequisite for establishing their structure–activity relationships and clinical utility [14].

*Tremella aurantialba*—treasured in China as the peer of ginseng and deer antler—was canonized in the *Compendium of Materia Medica* as the “golden ear that dissipates accumulations, soothes abdominal pain and heals incised wounds,” an early hint of immunomodulatory and anti-tumor activity [15,16]. *T. aurantialba* is widely regarded as the most nutritionally valuable of China’s “three jellied fungi” (*T. aurantialba*, *T. fuciformis*, *Auricularia auricula-judae*), a status likely attributable to its elevated heteropolysaccharide content [17]. Heteropolysaccharides from both fruiting bodies and mycelia exhibit potent bioactivities, most notably immunoregulation [18,19,20,21], and because activity is dictated by structure, obtaining structurally homogeneous fractions and defining their fine structures are prerequisites for any deeper exploration. Thus far, glucuronoxylomannan (GXM) is the sole well-defined isolate from *T. aurantialba*: an α-(1→3)-mannan (Man) backbone whose side-chain dress varies dramatically. Du’s TAPA1 sports xylose (Xyl) at *O*-4 and sporadic *O*-2 appendages of Xyl-Man-glucuronic acid (GlcA) or Xyl-Man; TAPB1 offers a di-Xyl prong at *O*-4 and Xyl-GlcA at *O*-2 [20,22]. Our own TAP-3 alternates α-(1→3)- with α-(1→2)-Man units, each *O*-2 graced with β-(1→4)-GlcA and β-(1→3)-Xyl terminated by β-Man, the whole lightly *O*-6-acetylated—an architecture that potently triggers nitric oxide (NO) and cytokine release from macrophages via toll-like receptor 4 (TLR4) [23]. Recently, a novel fucosylated glucuronoxylomannan was isolated from *T. aurantia* [24]. Beyond these GXMs, however, the structural landscape of *T. aurantialba* heteropolysaccharides remains largely uncharted; systematic isolation and rigorous characterization of further homogeneous fractions are therefore essential if this medicinal resource is to be fully exploited.

Herein, we describe the purification and structural dissection of a hitherto uncharacterized glycan, TAP-2a, from *T. aurantialba* fruiting bodies. Monosaccharide analysis, molecular weight (*M*_w_) determination, Fourier-transform infrared spectroscopy (FT-IR), methylation/GC–MS, and multidimensional NMR defined its covalent architecture, and immunological profiling in RAW264.7 macrophages provides an initial gauge of its translational potential as a macrophage-directed immunomodulator.

## 2. Materials and Methods

### 2.1. Materials and Reagents

Dried fruiting bodies of *T. aurantialba* were procured from the Kunming Wild Fungus Market (Kunming, China). Sepharose CL-6B and DEAE Sepharose Fast Flow were obtained from GE Healthcare (Uppsala, Sweden). 3-Methyl-1-phenyl-2-pyrazolin-5-one (PMP), lipopolysaccharide (LPS) from *E. coli* O111:B4, and monosaccharide standards—ᴅ-glucose (ᴅ-Glc), ᴅ-galactose, ᴅ-GlcA, ᴅ-galacturonic acid (ᴅ-GalA), and ʟ-rhamnose—were supplied by Sigma-Aldrich (St. Louis, MO, USA). ᴅ-Ribose (ᴅ-Rib), ᴅ-Man, ʟ-arabinose (ʟ-Ara), ᴅ-Xyl, and ʟ-fucose (ʟ-Fuc) were sourced from Aladdin Chemical Reagent Co., Ltd. (Shanghai, China). Fetal bovine serum (FBS), Dulbecco’s Modified Eagle Medium (DMEM), and the penicillin–streptomycin mixture were provided by Gibco (Grand Island, NY, USA). Enzyme-linked immunosorbent assay (ELISA) kits for mouse interleukin-6 (IL-6), interleukin-1β (IL-1β), and tumor necrosis factor-α (TNF-α) were purchased from NeoBioscience (Shenzhen, China). The endotoxin assay kit was from Xiamen Bioendo Technology Co., Ltd. (Xiamen, China). All other chemicals and reagents were of analytical grade.

### 2.2. Isolation and Purification of Polysaccharide TAP-2a

Crude polysaccharide (TAP) was obtained from the dried fruiting bodies of *T. aurantialba* as previously reported [23]. The extract was applied to a DEAE-Sepharose Fast Flow column (40 × 2.6 cm) and stepwise-eluted with 0–0.5 M NaCl; the fraction released at 0.1 M NaCl was collected, desalted, concentrated, and lyophilized. The resulting material was further purified on a Sepharose CL-6B column (150 × 2.0 cm) eluted with 0.2 M NaCl. Carbohydrate-positive fractions (phenol–sulfuric acid assay) were pooled, dialyzed against distilled water (3 d, 3.5 kDa MWCO), and lyophilized to afford the homogeneous polysaccharide TAP-2a.

### 2.3. Physicochemical Characterization of TAP-2a

Homogeneity and apparent *M*_w_ of TAP-2a were determined by high-performance gel-permeation chromatography (HPGPC) on a Shodex OHpak SB-804 HQ column (8 × 300 mm) coupled to a RID detector (Shimadzu, Kyoto, Japan). The column was eluted with 0.1 M NaCl at 0.5 mL/min and calibrated with a series of dextran standards (5.25–344 kDa). The presence of protein or nucleic acid contaminants was assessed by UV–Vis spectroscopy (Evolution 350, Thermo Fisher, Waltham, MA, USA) over the 190–800 nm range; absence of absorbance at 260 and 280 nm confirmed their removal. The level of endotoxin in TAP-2a was measured using an Endotoxin assay kit according to the manufacturer’s instruction.

### 2.4. Structural Characterization of TAP-2a

#### 2.4.1. Monosaccharide Composition

Monosaccharide composition was determined by PMP pre-column derivatization [25]. Briefly, the polysaccharide was first hydrolyzed in 4 M trifluoroacetic acid (TFA) at 120 °C for 2 h. After hydrolysis, the residual TFA was completely removed by repeated rotary evaporation with methanol (three cycles). The dried hydrolysate was re-dissolved in deionized water and then subjected to PMP derivatization under alkaline conditions at 70 °C for 100 min. After neutralization with 0.3 M HCl and chloroform extraction, the aqueous phase was analyzed on a Shimadzu LC-2030C 3D HPLC (Shimadzu Corp., Kyoto, Japan) (Eclipse Plus C18, 4.6 × 250 mm, 30 °C; 245 nm; PBS-CH_3_CN 83:17, 1 mL/min). Peak identities and molar ratios were assigned by comparing retention times and peak areas with calibration curves constructed from authentic standards of Man, Rib, Rha, GlcA, GalA, Glc, Gal, Ara, Xyl, and Fuc.

#### 2.4.2. FT-IR Spectroscopy

Functional groups were identified on a Nicolet iS50 FT-IR spectrometer (Thermo Fisher). TAP-2a (1 mg) was ground with dry KBr (100 mg) and pressed into a 1 mm pellet. The transmission spectrum was recorded from 4000 to 400 cm^−1^ at 4 cm^−1^ resolution (32 scans).

#### 2.4.3. Methylation Analysis

Methylation was carried out according to the classical procedure of Ciucanu and Kerek [26]. Briefly, 5 mg of the dry polysaccharide was first solubilized in anhydrous dimethyl sulfoxide (DMSO) under a gentle stream of nitrogen. Freshly ground NaOH was then introduced to generate the alkoxide, and methyl iodide was added drop-wise to effect permethylation. The entire sequence was repeated until FT-IR examination of an aliquot showed complete disappearance of the O–H stretching band at ≈3400 cm^−1^, signifying that every free hydroxyl group had been converted into its methyl ether. The fully methylated derivative was subsequently subjected to acid hydrolysis to cleave the glycosidic linkages, and the resulting partially methylated monosaccharides were reduced with NaBH_4_ and acetylated with acetic anhydride to yield the corresponding partially methylated alditol acetates (PMAAs). PMAAs were separated on an Agilent 7890B-5977B GC–MS system fitted with an HP-5 MS fused-silica capillary column (30 m × 0.25 mm × 0.25 μm). Injector 260 °C; oven 50 °C (1 min)→130 °C (50 °C/min)→230 °C (3 °C/min, hold 2 min); He, 1.0 mL/min.

#### 2.4.4. NMR Spectroscopy

TAP-2a (30 mg) was dissolved in 0.5 mL deuterium oxide (D_2_O, 99.9%). After centrifugation, the supernatant was collected and lyophilized. The freeze–thaw cycle was repeated three times to ensure complete hydrogen exchange. The finally dried sample was redissolved in 0.5 mL of fresh D_2_O and immediately analyzed. All one- and two-dimensional NMR experiments were recorded at 298 K on a Bruker AVANCE III HD 600 MHz spectrometer (Bruker BioSpin GmbH, Ettlingen, Germany) equipped with a 5 mm TCI cryoprobe and used to acquire 1D and 2D spectra: ^1^H, ^13^C, DEPT-135, ^1^H–^1^H COSY, ^1^H–^1^H TOCSY, ^1^H–^1^H ROESY, ^1^H–^13^C HSQC, ^1^H–^13^C HMBC, and ^1^H–^13^C HSQC-TOCSY.

### 2.5. Immunostimulatory Activity of TAP-2a

#### 2.5.1. Cell Viability Assay

RAW264.7 mouse macrophage cells were maintained in DMEM supplemented with 10% (*v*/*v*) FBS and 1% (*v*/*v*) penicillin–streptomycin at 37 °C in a humidified atmosphere containing 5% CO_2_. To evaluate the cytotoxicity of TAP-2a, cells were seeded in 96-well plates and allowed to adhere for 12 h. The medium was then replaced with fresh complete medium containing TAP-2a at 15, 30, 60, 120, or 240 μg/mL; control wells received medium alone (blank) or 1 μg/mL LPS (positive control). After 24 h incubation, 20 μL of 5 mg/mL MTT solution was added to each well, and the plates were returned to the incubator for 4 h. The resulting formazan crystals were dissolved in 150 μL DMSO, and absorbance was read at 490 nm on a VICTOR Nivo microplate reader (PerkinElmer, Waltham, MA, USA). Cell viability was expressed as the percentage of the blank control.

#### 2.5.2. Phagocytosis Assay

RAW264.7 cells were seeded as above and exposed to TAP-2a (or controls) for 24 h. FITC-dextran (1 mg/mL in complete medium) was then added, and the plates were incubated for a further 1 h at 37 °C. Wells were washed three times with ice-cold PBS to remove unincorporated dextran, and cells were resuspended in PBS. FITC fluorescence was quantified immediately on a BD Accuri™ C6 Plus flow cytometer (BD Biosciences, San Jose, CA, USA).

#### 2.5.3. Nitric Oxide (NO) Determination

Culture supernatants from the 24 h treatment plates were mixed with an equal volume of Griess reagent A (1% sulphanilamide in 5% phosphoric acid), followed by Griess reagent B (0.1% N-(1-naphthyl)ethylenediamine dihydrochloride). After 10 min at room temperature in the dark, absorbance was measured at 540 nm. Nitrite concentration was calculated from a NaNO_2_ standard curve (1–100 μM) and expressed as μM NO_2_^−^.

#### 2.5.4. Cytokine Secretion

Supernatants collected after 24 h stimulation were centrifuged (400× *g*, 5 min) to remove cellular debris. Levels of TNF-α, IL-1β, and IL-6 were determined with commercial ELISA kits used strictly according to the manufacturer’s instructions. Standard curves were run on each plate; results are presented as pg/mL after subtraction of background.

#### 2.5.5. Quantitative Real-Time PCR (qRT-PCR)

RAW264.7 cells were incubated in 6-well plates overnight and then treated with TAP-2a, LPS (1 μg/mL), or culture medium for 24 h. Total RNA was isolated with TRIzol^®^ reagent (Invitrogen, Carlsbad, CA, USA) and quantified spectrophotometrically. The RNA was reverse-transcribed using HiScript III RT SuperMix for qPCR (+gDNA wiper) (Vazyme Biotech, Nanjing, China). qRT-PCR was performed on a LightCycler^®^ 480 system (Roche, Basel, Switzerland) with SYBR Green Master Mix (Vazyme) in 10 μL reactions containing 200 nM gene-specific primers (Table S1). GAPDH served as the endogenous reference, and relative expression was calculated by the 2^−ΔΔCt^ method.

### 2.6. Statistical Analysis

The IBM SPSS Statistics 26 software was employed to conduct statistical analyses, which involved a one-way ANOVA followed by Duncan’s multiple comparisons. The level of statistical significance was determined at *p* < 0.05, and the data are displayed as the mean ± standard deviation (SD).

## 3. Results and Discussion

### 3.1. Isolation and Purification of TAP-2a

**Figure 1:**
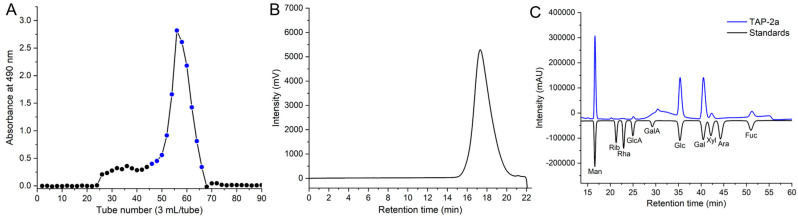
Purification and physicochemical characterization of TAP-2a. (**A**) Sepharose CL-6B size-exclusion profile of TAP-2 (blue section: pooled TAP-2a); (**B**) HPGPC chromatogram; (**C**) monosaccharide composition.

The crude polysaccharide from *T. aurantialba* was fractionated on DEAE-Sepharose Fast Flow into three peaks: TAP-1, TAP-2, and TAP-3. The structure of TAP-3 has been described previously [23]; TAP-1 will be further investigated in subsequent research. TAP-2 was rechromatographed on Sepharose CL-6B, affording a single, symmetrical peak (TAP-2a; Figure 1A,B) that appeared homogeneous and highly pure. HPGPC estimated its *M*_w_ at 16.95 kDa, and the UV trace (Figure S1) lacked 260/280 nm absorbance, confirming the absence of protein and nucleic acids. Using the endotoxin assay kit, the endotoxin content in TAP-2a was determined to be 0.00133 ± 0.000028 EU/µg, indicating that the presence of endotoxin in TAP-2a is negligible [14,27].

### 3.2. Monosaccharide Compositions

PMP pre-column derivatization showed TAP-2a to contain Man, GlcA, Glc, Gal, Xyl, and Fuc in a molar ratio of 5.57:1.00:9.90:6.29:1.31:2.47 (Figure 1C). Its monosaccharide profile and molar ratio differ sharply from those of all heteropolysaccharides previously reported for *T. aurantialba*. The acidic fraction TAPA1 described by Du et al. contains Man, Xyl, and GlcA at ≈5:4:1, with trace GalA and Glc; TAPB1 shows Man:Xyl:GlcA = 3.1:2.9:1.2 plus minor Gal, Glc, and GalA [20,22]. Our earlier TAP-3 gave Man:Xyl:GlcA = 3.0:1.0:1.0 [23]. Polysaccharides from *T. fuciformis* are likewise Man- and Xyl-rich, whereas the GXM-type AAPS of *A. auricula-judae* is dominated by Man (65%) together with GlcA (15%), Xyl (10%), and small amounts of Gal and Glc, and additionally, TMPA and TMPB obtained from *T. mesenterica* are composed of Xyl, Man, and Glc, with molar ratios of 1.00:4.74:0.91 and 1.00:6.63:2.34, respectively [28,29,30]. To our knowledge, no naturally occurring heteropolysaccharide exhibiting the monosaccharide composition of TAP-2a has been documented.

### 3.3. FT-IR

The FT-IR trace Figure S2, displayed typical polysaccharide signals: broad O–H stretch at ≈3400 cm^−1^, C–H stretch at ≈2930 cm^−1^, which corresponds to the stretching vibrations of O-H and C-H, respectively [31], and H_2_O bending at ≈1650 cm^−1^ [32]. Bands at 1131 cm^−1^, 1072 cm^−1^, and 1046 cm^−1^ arise from C–O bending in pyranose rings [33], while the sharp bands at 806 cm^−1^ and 890 cm^−1^ indicate α- and β-glycosidic linkages, respectively [34,35].

### 3.4. Methylation Analysis

**Figure 2:**
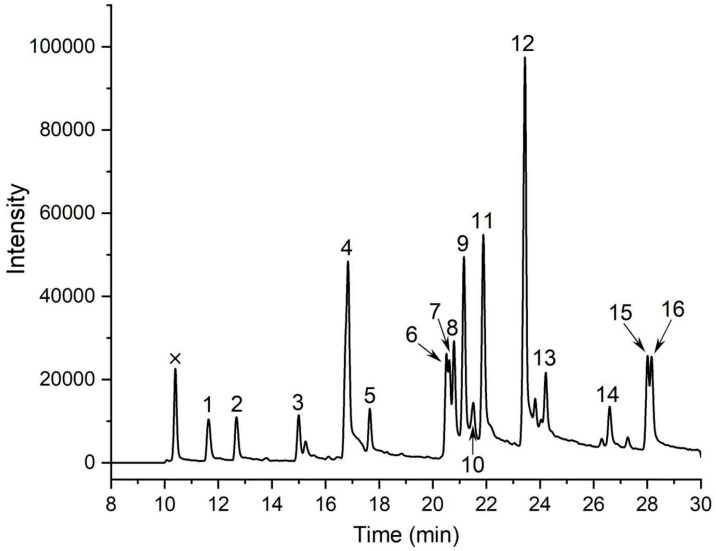
GC–MS chromatogram of PMAAs derived from TAP-2a. Peaks: 1, Xyl-(1→; 2, Fuc-(1→; 3, →4)-Xyl-(1→; 4, Glc-(1→; 5, Gal-(1→; 6, →2)-Man-(1→; 7, →3)-Glc-(1→; 8, →4)-Gal-(1→; 9, →3)-Man-(1→; 10, →3)-Gal-(1→; 11, →6)-Glc-(1→; 12, →6)-Gal-(1→; 13, →2,3)-Man-(1→; 14, →3,6)-Glc-(1→; 15, →2,6)-Gal-(1→; 16, →3,6)-Gal-(1→. Non-carbohydrate signals are labeled with an ×.

Methylation followed by GC–MS is the gold standard for mapping glycosidic linkages. TAP-2a was permethylated, hydrolyzed, reduced, and acetylated; the resulting 16 PMAAs were identified by retention time, fragment ions (Figure 2 and Figure S3), and the CCRC database. The data (Table 1) reveal a highly branched architecture: Glc is present as t-Glc (14.90%), →6)-Glc-(1→(11.51%), →3)-Glc-(1→(3.27%), and →3,6)-Glc-(1→(2.91%); mannose as →3)-Man-(1→(10.32%), →2)-Man-(1→(3.91%), and →2,3)-Man-(1→(3.91%); galactose as →6)-Gal-(1→(20.71%), →4)-Gal-(1→(5.44%), →3,6)-Gal-(1→(4.45%), →2,6)-Gal-(1→(4.31%), →3)-Gal-(1→(3.60%), and t-Gal (2.78%). Minor residues are t-Xyl (2.84%), →4)-Xyl-(1→(2.44%), and t-Fuc (2.68%). These results underscore the structural complexity of TAP-2a.

### 3.5. NMR Analysis of TAP-2a

**Figure 3:**
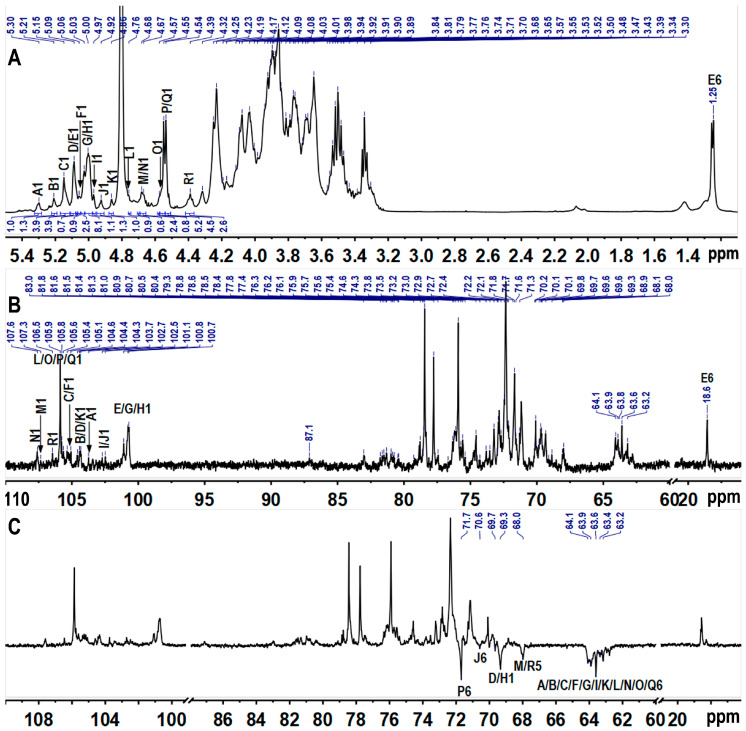
^1^H (**A**), ^13^C (**B**), and DEPT-135 (**C**) spectra. Labels are the same as those described in Table 2.

**Figure 4:**
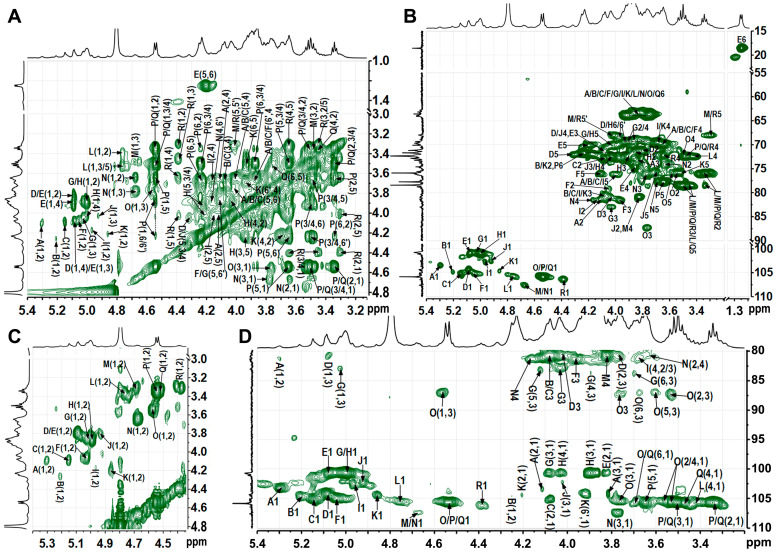
^1^H−^1^H TOCSY spectrum (**A**), ^1^H−^13^C HSQC spectrum (**B**), partial ^1^H−^1^H COSY spectrum (**C**), and partial ^1^H−^13^C HSQC-TOCSY spectrum (**D**). Labels are the same as those described in Table 2.

**Figure 5:**
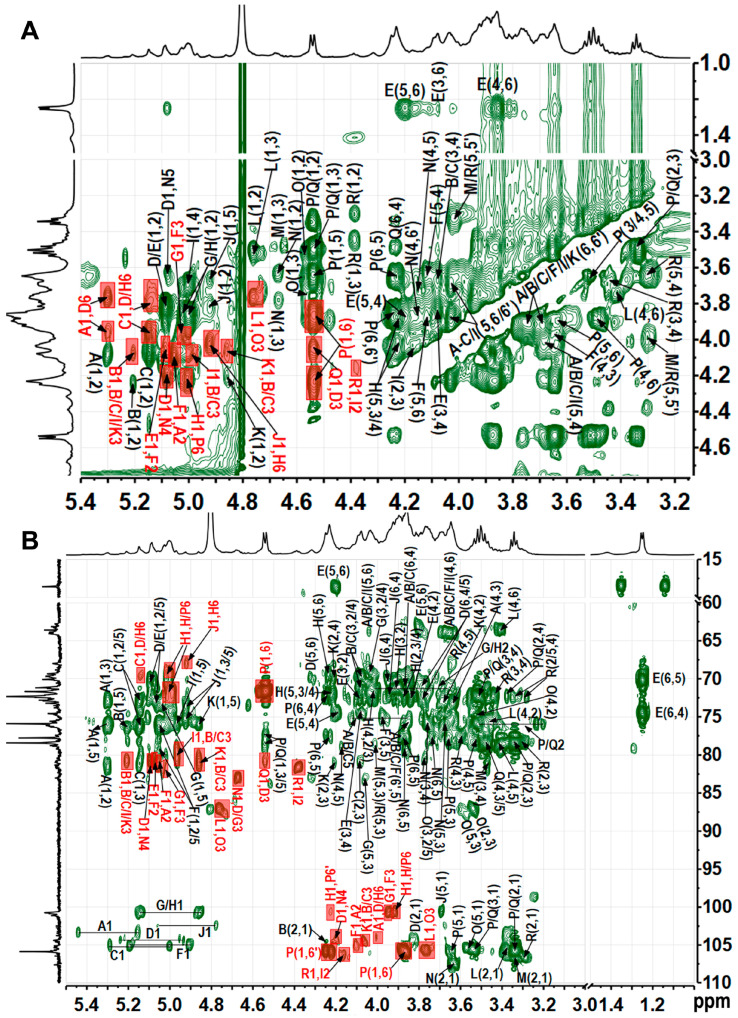
^1^H−^1^H ROESY spectrum (**A**), ^1^H−^13^C HMBC spectrum (**B**) of TAP-2a. Labels are the same as those described in Table 2.

Complete 1D and 2D NMR datasets (^1^H, ^13^C, COSY, HSQC, HMBC, NOESY) (Figure 3, Figure 4 and Figure 5) were acquired to refine the TAP-2a structure. Anomeric protons resonated between δ 4.35 and 5.45 ppm, and the corresponding carbons appeared at δ 100–108 ppm, evidencing a mixed α/β configuration [36]. HSQC cross-peaks allowed resolution of 18 discrete anomeric centres: δ_H_/δ_C_ 5.30/103.7 (A), 5.21/104.4 (B), 5.15/105.4 (C), 5.09/104.3 (D), 5.08/101.1 (E), 5.06/105.1 (F), 5.03/100.8 (G), 5.00/100.7 (H), 4.97/102.7 (I), 4.92/102.5 (J), 4.86/104.6 (K), 4.76/105.6 (L), 4.68/107.3 (M), 4.67/107.6 (N), 4.57/105.8 (O), 4.54/105.9 (P/Q), 4.39/106.4 (R) (Figure 4B and Table 2). Residues A–I exhibit ^1^*J*_C–H_ > 171 Hz (α-anomers), whereas J–R display ^1^*J*_C–H_ < 169 Hz (β-anomers) [37]. DEPT-135 negative signals (Figure 1C) at δ_C_ 62.8–64.1 (C-6 of Glc/Gal/Man) and strong inverted peaks at δ_C_ 71.6, 69.3, 68.1 (plus weaker ones at 69.6, 70.5) confirm 6-substitutions in hexoses and C-5 of Xyl [38].

Based on the methylation data, the most intense signals in the ^1^H spectrum—such as those at δ 5.00 and 4.54 ppm—should originate from →6)-Gal*p*-(1→(H), →6)-Glc*p*-(1→(P), and Glc*p*-(1→(Q) residues, with the largest peak likely contributing to residue H. In the COSY spectrum, the cross-peaks δ_H/H_ 5.00/3.87 and 5.00/3.92 readily assign H-2 and H-3 of residue H; TOCSY correlations from δ_H/H_ 5.00/4.04, 3.92/4.23, and 4.23/4.03 then extend the sequence to H-4 and H-5. HSQC-TOCSY and HMBC show δ_H/C_ 4.23/69.3 (H-5/C-6), while HMBC adds δ_H/C_ 4.23/72.4 (H-5/C-3/4); together with HSQC, these data complete the ^1^H/^13^C assignment of H. Likewise, residue G is →3)-α-Gal*p*-(1→: HSQC-TOCSY δ 5.03/83.0 fixes C-3 at δ83.0, HSQC gives its H-3 at δ4.05, and further HSQC-TOCSY correlations of δ_H_ 3.90, 4.13, and 3.70 to δ_C_ 83.0 secure the full proton-carbon map of G. Residue D was likewise identified as →3,6)-α-Gal*p*-(1→by the downfield-shifted H-3/C-3 (δ_H/C_ 4.05/80.4) and H-6/C-6 (δ_H/C_ 3.76/3.99/69.7) pairs. All signals of sugar residues L, O, P, and Q were assigned from 2D spectra. According to relevant literature [39,40], with ROESY cross-peaks between H-1 and H-2, H-3 and H-5, and with most proton chemical shifts being small except at the linkage positions, the four units were identified as β-Glc*p*. In the TOCSY spectrum of P, H-1 (δ_H_ 4.54) correlates with H-6/H-6ʹ (δ_H_ 3.89 and 4.23); the corresponding C-6 at 71.7 ppm in HSQC establishes P as →6)-β-Glc*p*-(1→. For Q, H-6/C-6 at δ_H/C_ 3.69/3.80/63.6 indicates a terminal β-Glc*p*-(1→whose signals largely overlap those of P. The HSQC-TOCSY cross-peak at δ_H/C_ 4.57/87.1 gives the C-3 of O as 87.1 ppm, with its H-3 at δ_H_ 3.77 in HSQC; the marked downfield shift of H-3/C-3 identifies O as →3)-β-Glc*p*-(1→, and all other ring positions were readily traced in the same HSQC-TOCSY map. Residues L and Q were assigned as terminal β-Glc*p*-(1→units, likely attached to distinct sugar partners. Residues A–C, F, I, and K were assigned to α/β-ᴅ-Man*p* based on the characteristically deshielded H-2 signals (4.07–4.26 ppm) [29]. Residue A, exhibiting a downfield C-2 shift, was identified as →2)-Man*p*-(1→; the same criterion located residues B, C, F, I, and K at →3)-Man*p*-(1→or →2,3)-Man*p*-(1→linkages. Differentiating the anomeric configuration of individual mannose units is sometimes equivocal, yet inspection of the C-5/H-5 region provides a reliable guide: β-anomers display markedly up-field shifts relative to their α-counterparts. Mannose is frequently acetylated at *O*-2 or *O*-3 [41,42,43]; a singlet at δ ≈ 2.07 confirmed the presence of acetyl groups [44], but the corresponding cross-peaks are too weak to permit unambiguous localization of the acetate. Considering the results of methylation analysis and the methyl group signals at δ 1.24/18.3, residue E was deduced to be terminal Fuc [36]. Methylation analysis and literature comparison [23] identified residue M as →4)-β-Xyl*p*-(1→and residue R as terminal β-Xyl*p*-(1→: both contain only five carbons and exhibit the characteristic H-5/H-5′/C-5 triad at δ 3.30/4.02/68.0. The H-4/C-4 pair of M (δ 3.83/80.7) is markedly deshielded relative to that of R (δ 3.64/71.8), confirming that C-4 of M bears the glycosidic linkage. The low-intensity anomeric proton of residue J identified it as →2,6)-β-Gal*p*-(1→, with H-2/C-2 at δ 3.80/80.7 and H-6/C-6 at δ 3.86/3.94/70.6 confirming glycosylation at both *O*-2 and *O*-6, in full agreement with methylation analysis. Residue N was assigned as →4)-β-Gal*p*-(1→, its shift pattern mirroring that of the corresponding unit in well-defined plant pectins.

Sequential glycosidic linkages were dissected by ROESY and HMBC (Figure 5 and Table 2). A-D connection: ROESY (5.30→3.76/3.99 ppm) and HMBC (3.99/103.7 ppm) place A at *O*-6 of D. D-N connection: ROESY (5.09/4.19 ppm) and HMBC (4.19/104.3, 5.09/81.8 ppm) anchor D to *O*-4 of N. N-G connection: HMBC (4.67/83.0 ppm) fixes N to *O*-3 of G. G-F connection: ROESY (5.03/3.96 ppm) and HMBC (5.03/81.3, 3.96/100.8 ppm) locate G at *O*-3 of F. F-A connection: ROESY (5.06/4.12 ppm) and HMBC (4.12/105.1 ppm) return F to *O*-2 of A, closing the Man-Gal pentasaccharide ring, while Q branches off D *O*-3 (4.54/4.05 and 4.54/80.4 ppm) and E off F *O*-2 (5.08/4.05 and 5.08/79.3 ppm). A contiguous Gal-Glc domain stitched together by 1→6 linkages was also identified. ROESY cross-peaks at 4.92/3.92 ppm and the HMBC correlation at 4.92/69.3 ppm place J at *O*-6 of H. ROESY (5.00/4.23 ppm) combined with HMBC contacts at 5.00/69.0–71.7 ppm and 3.94/4.23/100.7 ppm reveal that H extends to *O*-6 of either another H or P, propagating the chain. Finally, ROESY signals at 4.55/3.89/4.23 ppm and HMBC correlations at 4.55/71.7 ppm and 3.89/4.23/105.9 ppm confirm that P cyclizes back onto its own *O*-6 position, establishing the branch point. ROESY (4.76/87.1 ppm) and HMBC (3.77/105.6 ppm) place L at *O*-3 of O; the latter’s low abundance precludes assignment of its own linkage. Weak ROESY (4.39/4.16 ppm) and HMBC (4.39/81.3, 4.16/106.4 ppm) contacts trace a minor branch of R to *O*-2 of →2,3)-α-Man*p*-(1→(I). All remaining connectivities were deduced by the same protocol (Table 2).

Collectively, TAP-2a is a mannoglucogalactan dominated by Gal*p*, Glc*p,* and Man*p* with minor Xyl*p* and Fuc*p*, and it exhibits a far more intricate glycosidic repertoire than any polysaccharide previously described from this species. All earlier isolates—whether from our own work or from others—are canonical GXM-type polymers: high-molecular-weight chains constructed on a →3)-α-Man*p*-(1→backbone that carries β-Xyl*p* and β-Glc*p*A side stubs together with variable levels of *O*-acetylation [20,22,23]. A recent study described a fucosylated glucuronoxylomannan from the fruiting bodies of T. aurantia that also belongs to the GXM-type family; it possesses a molecular weight of ~1178 kDa and a high Fuc content of 19% [24]. Although mannoglucogalactans have been reported in fungi such as *Penicillium erythromellis*, *Aspergillus versicolor*, and a hybrid mushroom (*PfloVv12* × *Volvariella volvacea* backcross), these molecules display distinctly different signatures—e.g., β-ᴅ-Gal*f* or α-ᴅ-Glc*p* units, or the repeating block -{→6)-[(t-β-Man*p*)-(1→2)]-α-Glc*p*-(1→{6)-α-Glc*p*}*_n_*-(1→[45,46,47]. While TAP-2a does contain a Gal*p*→Glc*p* domain linked through 1,6-glycosidic bonds, its Glc residues adopt the β-anomeric configuration, a feature that sets its overall architecture apart from all previously characterized mannoglucogalactans. Thus, TAP-2a not only departs from the GXM family but also enriches the structural diversity of polysaccharides derived from *T. aurantialba*.

### 3.6. Effect of TAP-2a on RAW264.7 Cell Viability

**Figure 6:**
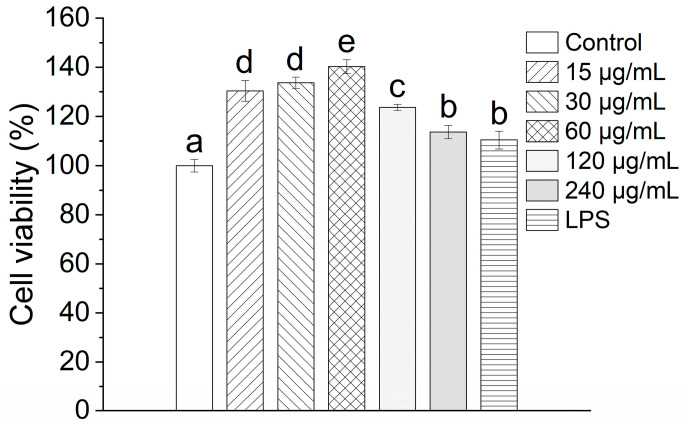
Cell viability of TAP-2a. Different lowercase letters (a–e) denote statistically significant differences (*p* < 0.05) among the tested groups.

The viability of RAW264.7 cells following exposure to TAP-2a was evaluated by means of the MTT assay. As depicted in Figure 6, the cells maintained robust proliferative activity after incubation with TAP-2a across the entire tested concentration range of 15–240 µg/mL, thereby demonstrating that TAP-2a exerts no discernible cytotoxicity toward RAW264.7 cells.

### 3.7. Effect of TAP-2a on Phagocytosis

**Figure 7:**
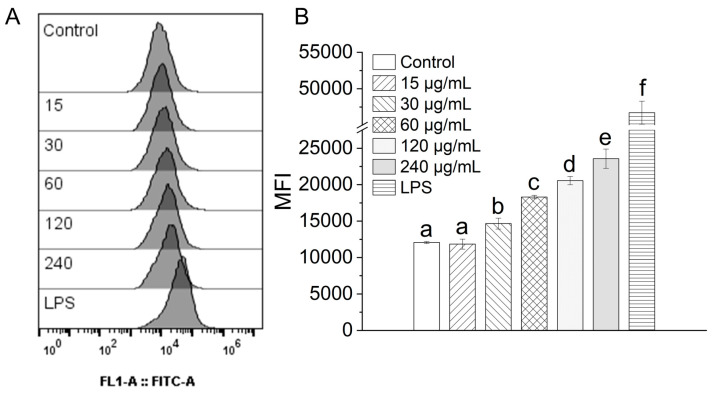
Influence of TAP-2a on the phagocytic capacity of RAW264.7 cells. (**A**) Representative flow-cytometry histograms illustrating FITC-dextran uptake in the control group, the LPS-positive control group (1 µg/mL), and the TAP-2a-treated groups (15–240 µg/mL). (**B**) MFI derived from the histograms serves as a quantitative index of phagocytic activity. Different lowercase letters (a–f) indicate statistically significant differences among the experimental groups (*p* < 0.05).

Upon activation, macrophages eradicate invading pathogens through phagocytosis—an indispensable process for initiating an effective immune response [48]. To evaluate the influence of TAP-2a on this critical function, phagocytic activity was quantified by flow cytometry using FITC-labeled dextran as a fluorescent probe, and the results are presented in Figure 7. Exposure to progressively higher concentrations of TAP-2a evoked a clear, dose-dependent elevation in mean fluorescence intensity (MFI). Specifically, RAW264.7 cells treated with TAP-2a at concentrations ranging from 30 to 240 μg/mL exhibited MFIs that were significantly elevated relative to those of the untreated control group, albeit still below the maximal response observed with the positive control (1 μg/mL LPS). Collectively, these findings demonstrate that TAP-2a is capable of markedly enhancing the phagocytic capacity of RAW264.7 macrophages when supplied at appropriate concentrations.

### 3.8. Effect of TAP-2a on the Production of NO and Cytokines

**Figure 8:**
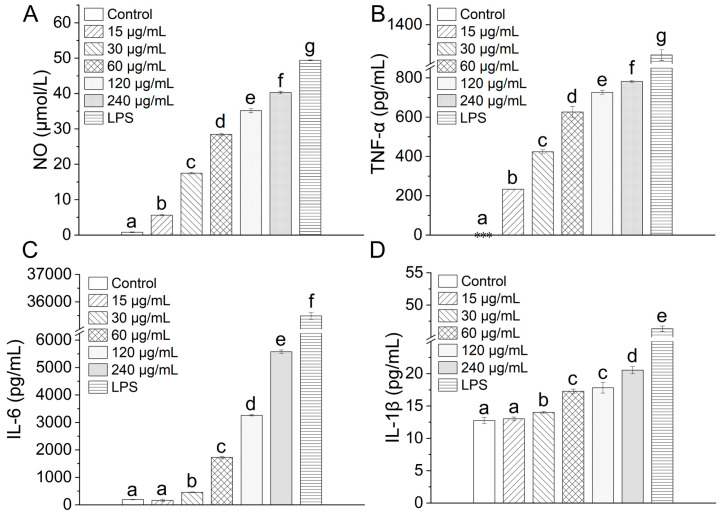
Inflammatory response induced by TAP-2a. (**A**–**D**) Secretion levels of NO, TNF-α, IL-6, and IL-1β. Different lowercase letters (a–g) denote statistically significant differences (*p* < 0.05) among groups. Low-expression groups are indicated by ***.

Upon encountering immunostimulatory agents such as polysaccharides, macrophages markedly up-regulate the generation of nitric oxide (NO) and a spectrum of cytokines that are indispensable for orchestrating effective immune responses [49,50]. To examine whether TAP-2a elicits such activation, NO released from RAW264.7 cells was quantified with the Griess reagent. As illustrated in Figure 8A, TAP-2a evoked a robust, dose-dependent elevation in NO secretion relative to the untreated control.

Subsequent ELISA analyses revealed that TAP-2a likewise modulates cytokine output. TNF-α production rose significantly above control levels and advanced progressively as the concentration of TAP-2a was escalated (Figure 8B). IL-1β and IL-6 exhibited similar dose-responsive profiles throughout the tested range (Figure 8C,D). At the lowest concentration evaluated (15 µg/mL), secretion of both IL-1β and IL-6 remained modest; however, concentrations from 30 to 240 µg/mL imparted a pronounced and statistically significant amplification of IL-1β and IL-6 release from RAW264.7 cells.

### 3.9. Effect of TAP-2a on the mRNA Expressions

**Figure 9:**
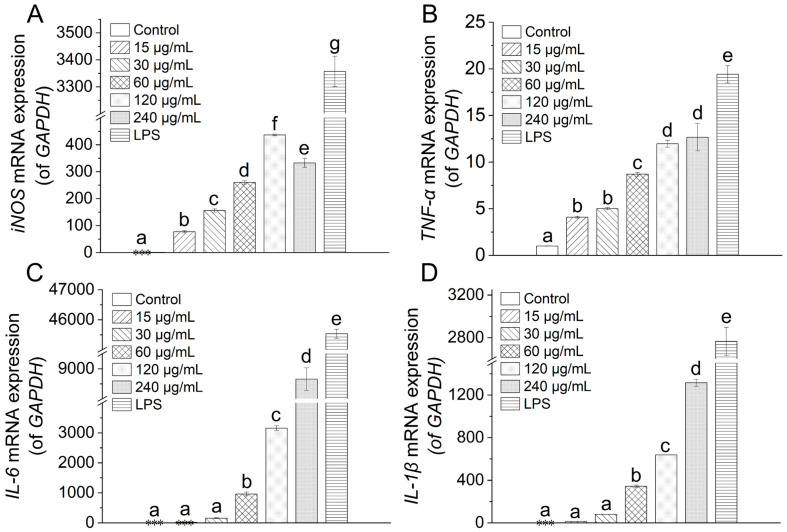
Inflammatory effects of TAP-2a. (**A**–**D**) Relative mRNA expression of iNOS, TNF-α, IL-6, and IL-1β. Different lowercase letters (a–g) indicate significant differences (*p* < 0.05) among groups. Low-expression groups are indicated by ***.

The synthesis of nitric oxide and cytokines is governed primarily by the transcriptional activity of their respective genes. To determine whether TAP-2a modulates these upstream events, we quantified the mRNA abundance of inducible nitric-oxide synthase (iNOS), TNF-α, IL-1β, and IL-6 in RAW264.7 macrophages by RT-qPCR after exposure to TAP-2a. Relative to the untreated control, TAP-2a evoked a pronounced, statistically significant up-regulation of iNOS and TNF-α transcripts at every concentration examined across the experimental range (Figure 9A,B). In contrast, significant elevation of IL-1β and IL-6 mRNA was only achieved at the higher concentrations of 60–240 µg/mL (*p* < 0.05) (Figure 9C,D). Taken together, these data corroborate the observed increases in NO and cytokine secretion and further support the conclusion that TAP-2a is capable of potentiating immune responsiveness in RAW264.7 macrophages through transcriptional activation of key pro-inflammatory genes.

Our findings demonstrate that TAP-2a is a potent immunomodulator, markedly enhancing the secretion and mRNA expression of NO, TNF-α, IL-6, and IL-1β. Similar to the clinically used mushroom polysaccharides lentinan, schizophyllan, and *Poria cocos* β-glucan, the receptors that TAP-2a may bind to, as well as the downstream signaling pathways, remain to be elucidated. Although its branched structure is more intricate than that of lentinan or schizophyllan, robust pre-clinical validation—pharmacokinetics, toxicology, and scale-up—will be required before TAP-2a can advance toward the clinic. Once fully characterized, it could follow the development path of *Astragalus* or ginseng polysaccharides [51], finding use as a botanical drug, functional food ingredient, or dietary supplement.

How chain length, branching density, side-chain make-up and length, and peripheral functionalities (carboxyl, acetyl, etc.) dictate the immunomodulatory potency of TAP-2a remains an open, worthwhile question [23]. Because earlier reports describe polysaccharides of widely divergent architectures, direct cross-study comparisons are necessarily inconclusive. Nevertheless, the available evidence suggests that high-molecular-weight heteroglycans—like the clinical β-glucans—can exert strong immune enhancement and merit continued development [14,23,24].

## 4. Conclusions

In this study, the novel heteropolysaccharide fraction TAP-2a was isolated from the fruiting bodies of *T. aurantialba* via sequential DEAE-Sepharose Fast Flow and Sepharose CL-6B chromatography. Based on monosaccharide composition, methylation analysis, and NMR spectroscopy, TAP-2a was elucidated as a new mannoglucogalactan that possesses a variety of glycosidic linkages, predominantly→6)-α-Gal*p*-(1→, →6)-β-Glc*p*-(1→, and →3)-α-Man*p*-(1→, and whose residues are interconnected in multiple modes, thereby generating a complex architecture distinct from those of previously reported polysaccharides. The mannoglucogalactan essentially comprises two distinct structural domains: one is built from Man*p* and Gal*p* and exhibits the repeating sequence -{→2)-α-Man*p*-(1→6)-[(t-β-Glc*p*)-(1→3)]-α-Gal*p*-(1→4)-β-Gal*p*-(1→3)-α-Gal*p*-(1→3)-[(t-α-Fuc*p*)-(1→2)]-α-Man*p*-(1-}*_n_*-, whereas the other is formed by Gal*p* and Glc*p* and displays the sequence -{→6)-α-Gal*p*}*_m_*-(1→{6)-β-Glc*p*}*_n_*-(1→; additionally, a fragment consisting of t-β-Glc*p*-(1→3)-β-Glc*p*-(1→ was identified, together with a minor branch in which β-Xyl is attached to *O*-2 of →2,3)-α-Man*p*-(1→. At the cellular level, TAP-2a exhibited clear immunoregulatory activity by enhancing phagocytosis and by up-regulating the production of nitric oxide, cytokines, and their corresponding mRNA transcripts. These findings underscore the potential of TAP-2a as a natural immunopotentiating agent and provide a comprehensive foundation for the further exploitation of *T. aurantialba*; nevertheless, additional investigations are required to clarify the detailed immunomodulatory mechanisms and structure–activity relationships of TAP-2a and to validate its immunological efficacy in vivo.

## Figures and Tables

**Table 1:** Glycosidic linkage composition of TAP-2a as determined by methylation–GC–MS analysis.

Peak No.	PMAAs	Molar Ratio	Deduced Residues
1	1,5-Di-*O*-acetyl-1-deuterio-2,3,4-tri-*O*-methyl-ᴅ-xylitol	2.84	Xyl-(1→
2	1,5-Di-*O*-acetyl-1-deuterio-6-deoxy-2,3,4-tri-*O*-methyl-ʟ-galactitol	2.68	Fuc-(1→
3	1,4,5-Tri-*O*-acetyl-1-deuterio-2,3-di-*O*-methyl-ᴅ-xylitol	2.44	→4)-Xyl-(1→
4	1,5-Di-*O*-acetyl-1-deuterio-2,3,4,6-tetra-*O*-methyl-ᴅ-glucitol	14.90	Glc-(1→
5	1,5-Di-*O*-acetyl-1-deuterio-2,3,4,6-tetra-*O*-methyl-ᴅ-galactitol	2.78	Gal-(1→
6	1,2,5-Tri-*O*-acetyl-1-deuterio-3,4,6-tri-*O*-methyl-ᴅ-mannitol	3.91	→2)-Man-(1→
7	1,3,5-Tri-*O*-acetyl-1-deuterio-2,4,6-tri-*O*-methyl-ᴅ-glucitol	3.27	→3)-Glc-(1→
8	1,4,5-Tri-*O*-acetyl-1-deuterio-2,3,6-tri-*O*-methyl-ᴅ-galactitol	5.44	→4)-Gal-(1→
9	1,3,5-Tri-*O*-acetyl-1-deuterio-2,4,6-tri-*O*-methyl-ᴅ-mannitol	10.32	→3)-Man-(1→
10	1,3,5-Tri-*O*-acetyl-1-deuterio-2,4,6-tri-*O*-methyl-ᴅ-galactitol	3.60	→3)-Gal-(1→
11	1,5,6-Tri-*O*-acetyl-1-deuterio-2,3,4-tri-*O*-methyl-ᴅ-glucitol	11.51	→6)-Glc-(1→
12	1,5,6-Tri-*O*-acetyl-1-deuterio-2,3,4-tri-*O*-methyl-ᴅ-galactitol	20.71	→6)-Gal-(1→
13	1,2,3,5-Tetra-*O*-acetyl-1-deuterio-4,6-di-*O*-methyl-ᴅ-mannitol	3.91	→2,3)-Man-(1→
14	1,3,5,6-Tetra-*O*-acetyl-1-deuterio-2,4-di-*O*-methyl-ᴅ-glucitol	2.91	→3,6)-Glc-(1→
15	1,2,5,6-Tetra-*O*-acetyl-1-deuterio-3,4-di-*O*-methyl-ᴅ-galactitol	4.31	→2,6)-Gal-(1→
16	1,3,5,6-Tetra-*O*-acetyl-1-deuterio-2,4-di-*O*-methyl-ᴅ-galactitol	4.45	→3,6)-Gal-(1→

**Table 2:** Chemical shift assignments of TAP-2a.

Residues	H/C	Chemical Shifts (δ, ppm)						Connected Residues and Sites
		1	2	3	4	5	6/6′	
→2)-α-Man*p*-(1→	H	5.30	**4.12**	3.81	3.65	4.00	3.74/3.88	
A	C	103.7	**81.5**	73.2	69.8	76.7	63.8	D6
→3)-α-Man*p*-(1→	H	5.21	4.25	**4.08**	3.64	4.02	3.74/3.88	
B	C	104.4	71.6	**80.7**	69.6	76.3	63.8	B/C/I/K3
→ 3)- α -Man*p*-(1→	H	5.15	4.08	**4.08**	3.63	4.00	3.74/3.88	
C	C	105.4	72.7	**80.5**	69.1	76.2	63.8	D/H6
→3,6)-α-Gal*p*-(1→	H	5.09	3.79	**4.05**	4.08	4.32	**3.76/3.99**	
D	C	104.3	71.3	**80.4**	71.3	72.1	**69.7**	N4
α-Fuc*p*-(1→	H	5.08	3.82	4.08	3.89	4.20	1.25	
E	C	101.1	72.2	71.3	74.3	70.1	18.6	F2
→2,3)-α-Man*p*-(1→	H	5.06	**4.05**	**3.96**	3.64	4.11	3.74/3.86	
F	C	105.1	**79.3**	**81.3**	69.8	76.2	63.4	A2
→3)-α-Gal*p*-(1→	H	5.03	3.84	**4.05**	3.90	4.13	3.70/3.83	
G	C	100.8	69.3	**83.0**	69.7	70.2	63.2	F3
→6)-α-Gal*p*-(1→	H	5.00	3.87	3.92	4.03	4.23	**3.76/3.92**	
H	C	100.7	71.7	72.3	72.4	69.7	**69.3**	P6
→2,3)-α-Man*p*-(1→	H	4.97	**4.16**	**4.08**	3.68	4.00	3.74/3.90	
I	C	102.7	**81.6**	**80.9**	69.1	76.2	63.9	B/C3
→2,6)-β-Gal*p*-(1→	H	4.92	**3.80**	4.03	4.07	3.69	**3.86/3.94**	
J	C	102.5	**80.7**	73.5	70.1	75.6	**70.6**	H6
→3)-β-Man*p*-(1→	H	4.86	4.20	**4.09**	3.68	3.51	3.71/3.92	
K	C	104.6	72.3	**80.7**	69.5	74.6	63.8	B/C3
β-Glc*p*-(1→	H	4.76	3.39	3.51	3.43	3.50	3.73/3.83	
L	C	105.6	76.1	78.8	72.3	78.4	64.1	O3
→4)-β-Xyl*p*-(1→	H	4.68	3.32	3.47	**3.83**	3.30/4.02		
M	C	107.3	76.1	78.6	**80.7**	68.1		n.d.
→4)-β-Gal*p*-(1→	H	4.67	3.64	3.79	**4.19**	3.65	3.73/3.85	
N	C	107.6	73.8	76.2	**81.8**	77.4	63.8	G3
→3)-β-Glc*p*-(1→	H	4.57	3.55	**3.77**	3.50	3.57	3.69/3.80	
O	C	105.8	75.4	**87.1**	72.4	75.7	63.8	M4
→6)-β-Glc*p*-(1→	H	4.55	3.34	3.50	3.52	3.65	**3.89/4.23**	
P	C	105.9	75.9	78.4	72.4	77.8	**71.7**	P6
β-Glc*p*-(1→	H	4.54	3.34	3.50	3.48	3.50	3.69/3.80	
Q	C	105.9	75.9	78.4	72.4	78.4	63.6	D3
β-Xyl*p*-(1→	H	4.39	3.31	3.46	3.64	3.30/4.02		
R	C	106.4	76.0	78.5	71.8	68.0		I2

Values in bold indicate glycosylated positions.

## Data Availability

The original contributions presented in this study are included in the article/Supplementary Material. Further inquiries can be directed to the corresponding authors.

## Supplementary Materials

The following supporting information can be downloaded at https://www.mdpi.com/article/10.3390/foods14234126/s1, Figure S1: UV spectrum of TAP-2a; Figure S2. FT-IR spectrum of purified TAP-2a; Figure S3: Mass spectra of PMAAs for the peaks shown in Figure 2; Table S1: Primer sequences for RT-qPCR.

## References

[1] Xie J.H., Jin M.L., Morris G.A., Zha X.Q., Yi Y., Li J.E., Wang Z.J., Gao J., Nie S.P., Shang P. (2016). Advances on Bioactive Polysaccharides from Medicinal Plants. Crit. Rev. Food Sci. Nutr..

[2] Wang W.L., Zhao B., Zhang Z.T., Kikuchi T., Li W., Jantrawut P., Feng F., Liu F.L., Zhang J. (2024). Natural Polysaccharides and Their Derivatives Targeting the Tumor Microenvironment: A Review. Int. J. Biol. Macromol..

[3] Wang P.C., Zhao S., Yang B.Y., Wang Q.H., Kuang H.X. (2016). Anti-Diabetic Polysaccharides from Natural Sources: A Review. Carbohydr. Polym..

[4] Yuan Q.X., Li H., Wang Q., Sun S.J., Fang Z.Y., Tang H., Shi X.H., Wen J., Huang L.H., Bai M. (2022). Deaminative-Cleaved *S. monotuberculatus* Fucosylated Glycosaminoglycan: Structural Elucidation and Anticoagulant Activity. Carbohydr. Polym..

[5] Schepetkin I.A., Quinn M.T. (2006). Botanical Polysaccharides: Macrophage Immunomodulation and Therapeutic Potential. Int. Immunopharmacol..

[6] Hou C.Y., Chen L.L., Yang L.Z., Ji X.L. (2020). An Insight into Anti-Inflammatory Effects of Natural Polysaccharides. Int. J. Biol. Macromol..

[7] Cai W.D., Wong K.H., Huang Q.L. (2023). Isolation, Structural Features, Rheological Properties and Bioactivities of Polysaccharides from *Lignosus rhinocerotis*: A Review. Int. J. Biol. Macromol..

[8] Liu J.X., Wang Y.X., Wu J.Z., Georgiev M.I., Xu B.J., Wong K.H., Bai W.B., Tian L.M. (2022). Isolation, Structural Properties, and Bioactivities of Polysaccharides from *Mushrooms termitomyces*: A Review. J. Agric. Food Chem..

[9] Ye S.Y., Gao Y., Hu X.Y., Cai J.Y., Sun S.W., Jiang J.H. (2024). Research Progress and Future Development Potential of *Flammulina velutipes* Polysaccharides in the Preparation Process, Structure Analysis, Biology, and Pharmacology: A Review. Int. J. Biol. Macromol..

[10] Barbosa J.R., de Carvalho Junior R.N. (2021). Polysaccharides Obtained from Natural Edible Sources and Their Role in Modulating the Immune System: Biologically Active Potential That Can Be Exploited against COVID-19. Trends Food Sci. Technol..

[11] Wasser S.P. (2002). Medicinal Mushrooms as a Source of Antitumor and Immunomodulating Polysaccharides. Appl. Microbiol. Biotechnol..

[12] Martel J., Ko Y.F., Ojcius D.M., Lu C.C., Chang C.J., Lin C.S., Lai H.C., Young J.D. (2017). Immunomodulatory Properties of Plants and Mushrooms. Trends Pharmacol. Sci..

[13] Wasser S.P. (2010). Medicinal Mushroom Science: History, Current Status, Future Trends, and Unsolved Problems. Int. J. Med. Mushrooms.

[14] Yuan Q.X., Liang R.Y., Lv K.L., Shi X.H., Leng J., Liu Y.H., Xiao J., Zhang L.F., Zhao L.Y. (2024). Structural Characterization of a *Chlorella heteropolysaccharide* by Analyzing Its Depolymerized Product and Finding an Inducer of Human Dendritic Cell Maturation. Carbohydr. Polym..

[15] Yan Y., Wang M., Chen N., Wang X., Fu C., Li Y., Gan X., Lv P., Zhang Y. (2022). Isolation, Structures, Bioactivities, Application and Future Prospective for Polysaccharides from *Tremella aurantialba*: A Review. Front. Immunol..

[16] Wang Z.X., Huang K., Pu K.L., Li L., Jiang W.X., Wu J., Kawagishi H., Li M.L., Qi J.Z. (2025). *Naematelia aurantialba*: A Comprehensive Review of Its Medicinal, Nutritional, and Cultivation Aspects. Food Med. Homol..

[17] Lo H.C., Tsai F.A., Wasser S.P., Yang J.G., Huang B.M. (2006). Effects of Ingested Fruiting Bodies, Submerged Culture Biomass, and Acidic Polysaccharide Glucuronoxylomannan of *Tremella mesenterica* Retz.:Fr. on Glycemic Responses in Normal and Diabetic Rats. Life Sci..

[18] Deng C., Sun Y.Y., Fu H.T., Zhang S.X., Chen J.H., Xu X. (2016). Antioxidant and Immunostimulatory Activities of Polysaccharides Extracted from *Tremella aurantialba* Mycelia. Mol. Med. Rep..

[19] Lee G.W., Hur H., Shim M.J., Lee T.S. (2008). Antitumor and Immune-Modulatory Effect of Drude Polysaccharides from Fruiting Body of *Tremella aurantialba* against Mouse Sarcoma 180. Korean J. Mycol..

[20] Du X.J., Zhang J.S., Yang Y., Ye L.B., Tang Q.J., Jia W., Liu Y.F., Zhou S., Hao R.X., Gong C.Y. (2009). Structural Elucidation and Immuno-Stimulating Activity of an Acidic Heteropolysaccharide (TAPA1) from *Tremella aurantialba*. Carbohydr. Res..

[21] Du X.J., Zhang J.S., Lv Z.W., Ye L.B., Yang Y., Tang Q.J. (2014). Chemical Modification of an Acidic Polysaccharide (TAPA1) from *Tremella aurantialba* and Potential Biological Activities. Food Chem..

[22] Du X.J., Zhang Y., Mu H.M., Lv Z.W., Yang Y., Zhang J.S. (2015). Structural Elucidation and Antioxidant Activity of a Novel Polysaccharide (TAPB1) from *Tremella aurantialba*. Food Hydrocoll..

[23] Yuan Q.X., Zhang X.D., Ma M.Y., Long T., Xiao C.L., Zhang J., Liu J.K., Zhao L.Y. (2020). Immunoenhancing Glucuronoxylomannan from *Tremella aurantialba* Bandoni et Zang and Its Low-Molecular-Weight Fractions by Radical Depolymerization: Properties, Structures and Effects on Macrophages. Carbohydr. Polym..

[24] Chen J., Ma Y., Rao Z.M., Jiang S.L., Lou Y.J., Malik K., Chowdhury A., Ying H.J., Yu C.H. (2025). A New Fucosylated Glucuronoxylomannan from the Fruit Bodies of *Tremella aurantia*: Structural Characterization and Immunoenhancing Activity on Seasonal Influenza mRNA Vaccine. Carbohydr. Polym..

[25] Yuan Q.X., Xie Y.F., Wang W., Yan Y.H., Ye H., Jabbar S., Zeng X.X. (2015). Extraction Optimization, Characterization and Antioxidant Activity *in Vitro* of Polysaccharides from Mulberry (*Morus alba* L.) Leaves. Carbohydr. Polym..

[26] Ciucanu I., Kerek F. (1984). A Simple and Rapid Method for the Permethylation of Carbohydrates. Carbohydr. Res..

[27] Tian S.S., Mao Z., Wang Y.X., Li K.W., Li Y.F., Zhu B.Q., Zhou F.M., Li J.C., Shen Y.Z., Ding Z.S. (2025). Structural Characterization and Immunomodulatory Activity Analysis of A Novel Pectic Polysaccharide Extracted from *Tetrastigma hemsleyanum* Diels et Gilg and Its Hydrolysis Products. Carbohydr. Polym..

[28] Wu Y.J., Wei Z.X., Zhang F.M., Linhardt R.J., Sun P.L., Zhang A.Q. (2019). Structure, Bioactivities and Applications of the Polysaccharides from *Tremella fuciformis* Mushroom: A Review. Int. J. Biol. Macromol..

[29] Perera N., Yang F.L., Chern J., Chiu H.W., Hsieh C.Y., Li L.H., Zhang Y.L., Hua K.F., Wu S.H. (2018). Carboxylic and: O -Acetyl Moieties Are Essential for the Immunostimulatory Activity of Glucuronoxylomannan: A Novel TLR4 Specific Immunostimulator from *Auricularia auricula-judae*. Chem. Commun..

[30] Yan Y.L., Yu C.H., Chen J., Li X.X., Wang W., Li S.Q. (2011). Ultrasonic-Assisted Extraction Optimized by Response Surface Methodology, Chemical Composition and Antioxidant Activity of Polysaccharides from *Tremella mesenterica*. Carbohydr. Polym..

[31] Flores-Morales A., Jiménez-Estrada M., Mora-Escobedo R. (2012). Determination of the Structural Changes by FT-IR, Raman, and CP/MAS 13C NMR Spectroscopy on Retrograded Starch of Maize Tortillas. Carbohydr. Polym..

[32] Liu X.P., Wang Q.Y., Wang J., Guo L., Chu Y.H., Ma C.Y., Kang W.Y. (2024). Structural Characterization, Chain Conformation and Immunomodulatory Activity of a Heteropolysaccharide from *Inonotus hispidus*. Int. J. Biol. Macromol..

[33] Chen G.J., Bai Y.X., Zeng Z.Q., Peng Y.J., Zhou W.T., Shen W.B., Zeng X.X., Liu Z.H. (2021). Structural Characterization and Immunostimulatory Activity of Heteropolysaccharides from Fuzhuan Brick Tea. J. Agric. Food Chem..

[34] Thambiraj S.R., Phillips M., Koyyalamudi S.R., Reddy N. (2018). Yellow Lupin (*Lupinus luteus* L.) Polysaccharides: Antioxidant, Immunomodulatory and Prebiotic Activities and Their Structural Characterisation. Food Chem..

[35] Ji X.L., Hou C.Y., Yan Y.Z., Shi M.M., Liu Y.Q. (2020). Comparison of Structural Characterization and Antioxidant Activity of Polysaccharides from Jujube (*Ziziphus jujuba* Mill.) Fruit. Int. J. Biol. Macromol..

[36] Yao H.Y.Y., Wang J.Q., Yin J.Y., Nie S.P., Xie M.Y. (2021). A Review of NMR Analysis in Polysaccharide Structure and Conformation: Progress, Challenge and Perspective. Food Res. Int..

[37] Cheng H.N., Neiss T.G. (2012). Solution NMR Spectroscopy of Food Polysaccharides. Polym. Rev..

[38] Sun T., Xu X.Y., Ma Y.H., Jiang H., Yang K., Wang R., Gu Y., Li S., Qiu Y.B., Sun D.F. (2023). Structure, Rheology, and Antifreeze Property of the Exopolysaccharide from *Naematelia aurantialba* through Basidiospore Fermentation. Food Hydrocoll..

[39] Samuelsen A.B.C., Rise F., Wilkins A.L., Teveleva L., Nyman A.A.T., Aachmann F.L. (2019). The Edible Mushroom *Albatrellus ovinus* Contains a α-L-Fuco-α-D-Galactan, α-D-Glucan, a Branched (1 → 6)-β-D-Glucan and a Branched (1 → 3)-β-D-Glucan. Carbohydr. Res..

[40] Ellefsen C.F., Wold C.W., Wilkins A.L., Rise F., Samuelsen A.B.C. (2021). Water-Soluble Polysaccharides from *Pleurotus eryngii* Fruiting Bodies, Their Activity and Affinity for Toll-like Receptor 2 and Dectin-1. Carbohydr. Polym..

[41] Hannuksela T., Du Penhoat C.H. (2004). NMR Structural Determination of Dissolved O-Acetylated Galactoglucomannan Isolated from Spruce Thermomechanical Pulp. Carbohydr. Res..

[42] Deng W.Q., Han S.W., Shao S.Y., Li S. (2025). Elucidation of the Fine Structure and Anti–Breast Tumor Activity of a Glucomannan from the Pseudobulbs of *Pleione bulbocodioides*. Carbohydr. Polym..

[43] Capek P., Alfo J., Liškova D. (2002). An Acetylated Galactoglucomannan from *Picea abies* L. *Karst*. Carbohydr. Res..

[44] Butt H.S., Ulriksen E.S., Rise F., Wangensteen H., Duus J.Ø., Inngjerdingen M., Inngjerdingen K.T. (2024). Structural Elucidation of Novel Pro-Inflammatory Polysaccharides from *Daphne mezereum* L. *Carbohydr*. Polym..

[45] Ruperez P., Leal J.A. (1987). Mannoglucogalactans from the Cell Walls of *Penicillium erythromellis*: Isolation and Partial Characterisation. Carbohydr. Res..

[46] Nandan C.K., Sarkar R., Bhanja S.K., Sikdar S.R., Islam S.S. (2011). Isolation and Characterization of Polysaccharides of a Hybrid Mushroom (Backcross Mating between *PfloVv12* and *Volvariella volvacea*). Carbohydr. Res..

[47] Yan M.X., Mao W.J., Liu X., Wang S.Y., Xia Z., Cao S.J., Li J., Qin L., Xian H.L. (2016). Extracellular Polysaccharide with Novel Structure and Antioxidant Property Produced by the Deep-Sea Fungus *Aspergillus versicolor* N2bc. Carbohydr. Polym..

[48] Carreras-Gonzalez A., Barriales D., Palacios A., Montesinos-Robledo M., Navasa N., Azkargorta M., Peña-Cearra A. (2019). Regulation of Macrophage Activity by Surface Receptors Contained within *Borrelia burgdorferi*-Enriched Phagosomal Fractions. PLoS Pathog..

[49] Tzianabos A.O. (2000). Polysaccharide Immunomodulators as Therapeutic Agents: Structural Aspects and Biologic Function. Clin. Microbiol. Rev..

[50] Li M.Z., Huang X.J., Wen J.J., Chen S.K., Wu X.C., Ma W.N., Cui S.W., Xie M.Y., Nie S.P. (2023). Innate Immune Receptors Co-Recognition of Polysaccharides Initiates Multi-Pathway Synergistic Immune Response. Carbohydr. Polym..

[51] Huang J., Liu D., Wang Y., Liu L., Li J., Yuan J., Jiang Z., Jiang Z., Hsiao W.W., Liu H. (2022). Ginseng polysaccharides alter the gut microbiota and kynurenine/tryptophan ratio, potentiating the antitumour effect of antiprogrammed cell death 1/programmed cell death ligand 1 (anti-PD-1/PD-L1) immunotherapy. Gut.

