# Supplementary material for: Structural Characterization and Immunomodulatory Activity of a Novel Mannoglucogalactan from Tremella aurantialba: Implications for Natural Immunotherapy
Source: Foods. 2025 Dec 2;14(23):4126. doi: 10.3390/foods14234126 (PMC12691847; doi:10.3390/foods14234126)
Supplement: Supplementary file 1 [file foods-14-04126-s001.zip › foods-3962951-supplementary.pdf]

Supplementary data

Structural characterization and immunomodulatory activity of a novel mannoglucogalactan from *Tremella aurantialba*: Implications for natural immunotherapy

Yuemou Zhao [†], Wenyu Liang [†], Huaqun Chen, Jinwen Huang, Longyan Zhao *, Qingxia Yuan *

Guangxi Key Laboratory of Marine Drugs, University Engineering Research Center of High-efficient Utilization of Marine Traditional Chinese Medicine Resources, Guangxi, Institute of Marine Drugs, Guangxi University of Chinese Medicine, Nanning 530200, China

* Corresponding author.

[†] These authors contributed equally to this work.

E-mail address: zhaolongyan-dra@163.com (L.Z.); qingxiayuan@163.com (Q.Y.)

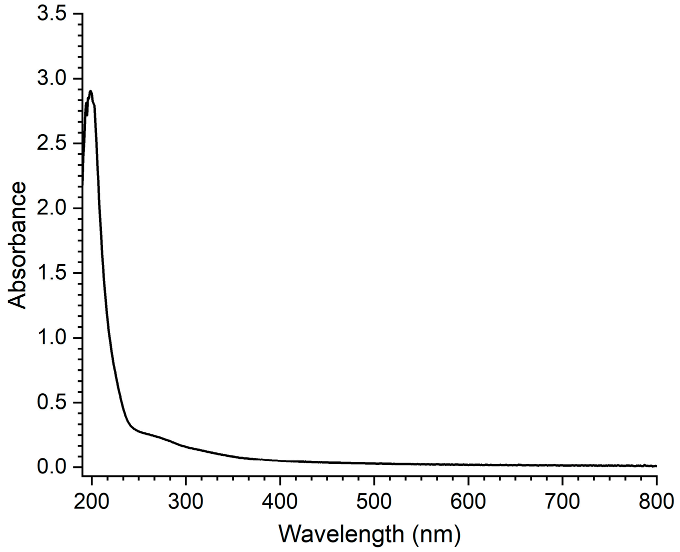

Figure S1. UV spectrum of TAP-2a.

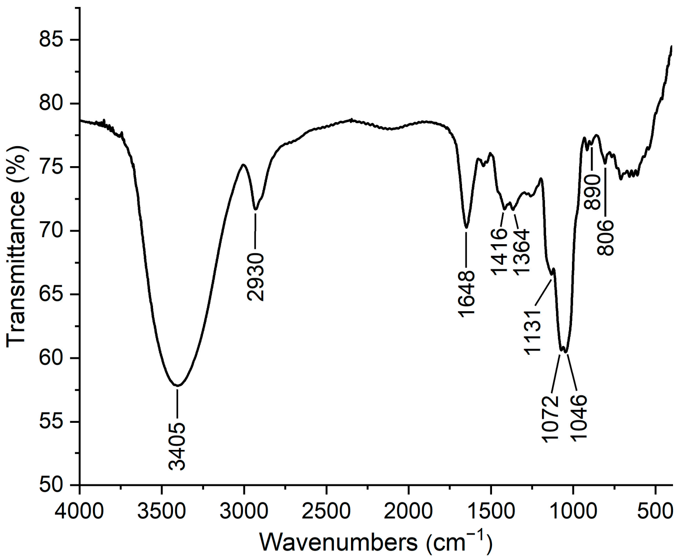

Figure S2. FT-IR spectrum of purified TAP-2a

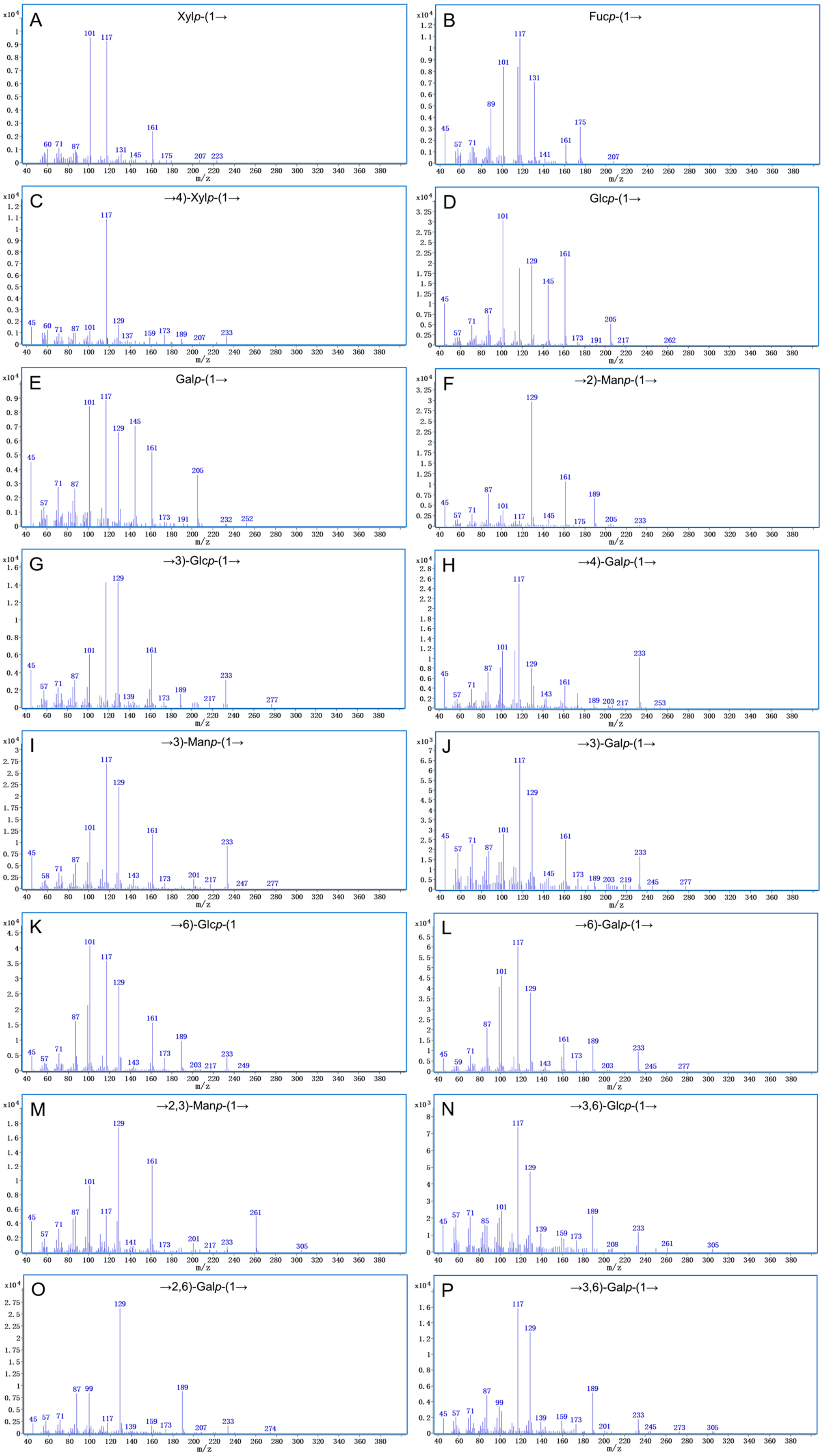

Figure S3. Mass spectra of PMAAs for the peaks shown in Fig. 2.

Table S1. Primer sequences for RT-qPCR

Primer Name	Primer Sequence	
GAPDH	Forward	5'-CAGAAGACTGTGGATGGCCC-3'
	Reverse	5'-ATCCACGACGGACACATTGG-3'
IL-1β	Forward	5'-GGTGTGTGACGTTCCCATTA-3'
	Reverse	5'-ATTGAGGTGGAGAGCTTTCAG-3'
iNOS	Forward	5'-GTTCTCAGCCCAACAATACAAGA-3'
	Reverse	5'-GTGGACGGGTCGATGTCAC-3'
TNF-α	Forward	5'-AGGGGATTATGGCTCAGGGT-3'
	Reverse	5'-CCACAGTCCAGGTCACTGTC-3'
IL-6	Forward	5'-GCCTTCTTGGGACTGATGCT-3'
	Reverse	5'-GACAGGTCTGTTGGGAGTGG-3'
